# The intersection of mitochondria, lipids, and ferroptosis: a new avenue for dry age-related macular degeneration

**DOI:** 10.1007/s10495-025-02165-2

**Published:** 2025-08-21

**Authors:** Jacob Dohl, Gordon Burns, Mithalesh Singh

**Affiliations:** 1 Department of Pathology & Laboratory Medicine, University of California, Irvine, CA 92697 USA; 2 Department of Ophthalmology, University of Texas Southwestern Medical Center, Dallas, TX USA

**Keywords:** Mitochondria, Age-related macular degeneration, Ferroptosis, Lipids, Reactive oxygen species, Retina

## Abstract

Age-related macular degeneration (AMD) is currently the leading cause of vision loss in developed countries. Despite decades of research and development, there are currently no treatments for the dry version of the illness. Dry AMD (DAMD) is a multifactorial disease stemming from dysfunction in the complement system, mitochondrial function, and lipid metabolism. While the complement system has been studied in-depth for its involvement in DAMD, mitochondria and lipids are understudied for their potential contributions to this process. Ferroptosis, an iron-dependent cell death mechanism, is associated with mitochondrial dysfunction and lipid dysregulation, and has been implicated as a driver of DAMD. This review describes the pathology of DAMD and the potential role of mitochondria, metabolism, and lipid dysregulation in the disease. We will highlight the intersection of pathways involving mitochondria, lipid dysregulation, and ferroptosis in DAMD progression, as well as the need for future studies to elucidate this connection.

**Graphical abstract:**

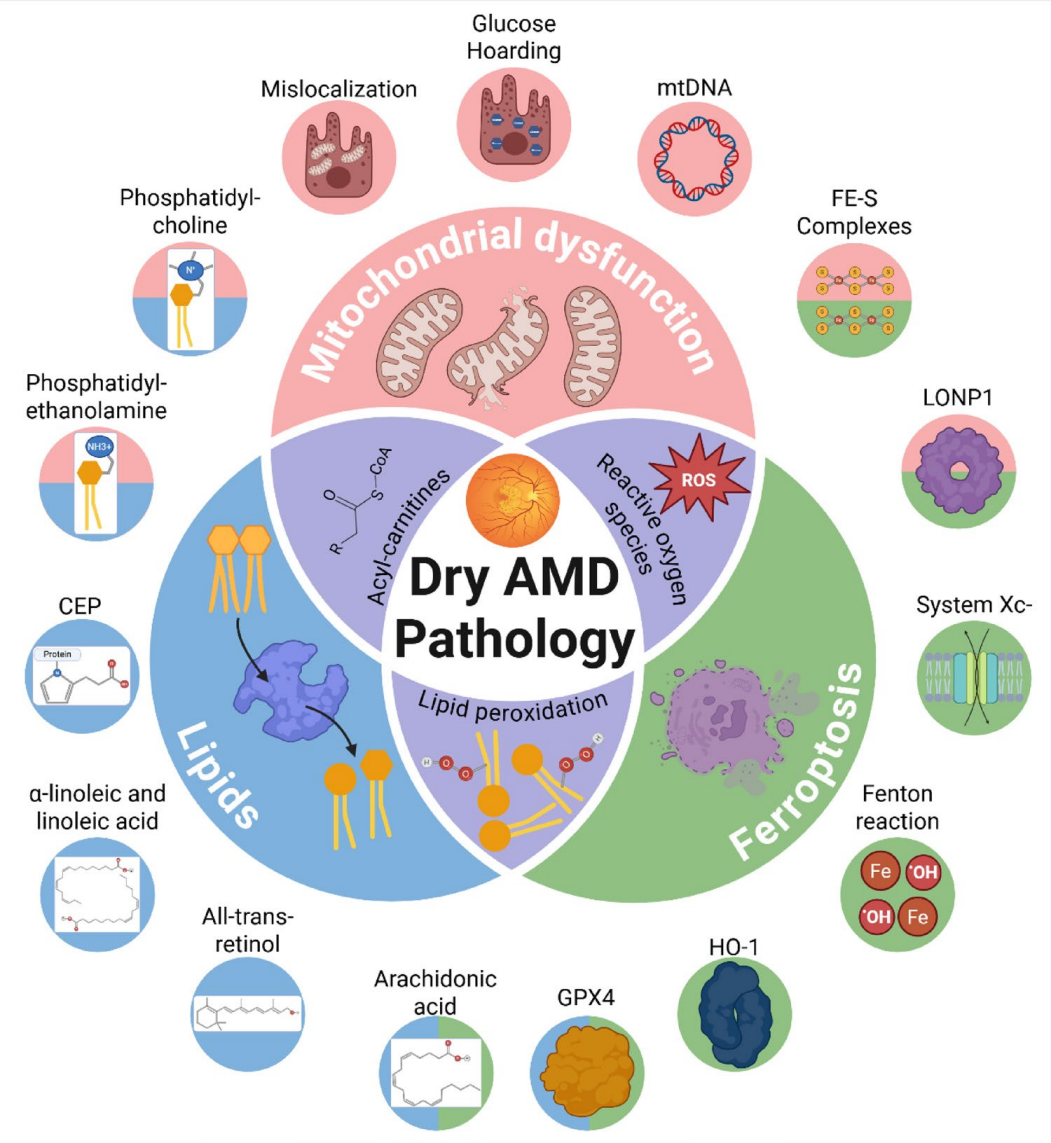

## Introduction

Age-related macular degeneration (AMD) is a leading cause of vision loss globally, affecting ~ 150 million people in 2020, with that number expected to increase by nearly 50% by 2040 [1, 2]. AMD involves the gradual loss of the macula, a central region of the human retina that contains both the fovea and most of the retinal cones, making it responsible for acute color vision [3, 4]. Macular degeneration is typically divided into two categories based on disease pathology: neovascular AMD and dry AMD (DAMD).

Neovascular AMD, also known as wet AMD, is characterized by the infiltration of choroidal vasculature into the retina, resulting in vascular leakage, retinal detachment, and macular degeneration [5]. It is the least common form of AMD, representing only ~ 10% of AMD cases; however, it also has the worst prognosis, with ~ 80–90% of patients becoming blind post-diagnosis, barring medical intervention [6–8]. Patients with wet AMD demonstrate increased vascular endothelial growth factor (VEGF) within the choroidal vasculature, prompting neovascularization; therefore, treatment for wet AMD is centered on anti-VEGF drugs, including brolucizumab, ranibizumab, and KSI-301 [9–12]. Early detection and treatment of wet AMD is often successful at inhibiting or preventing vision loss, with some patients regaining visual acuity following treatment [13].

**Fig. 1:**
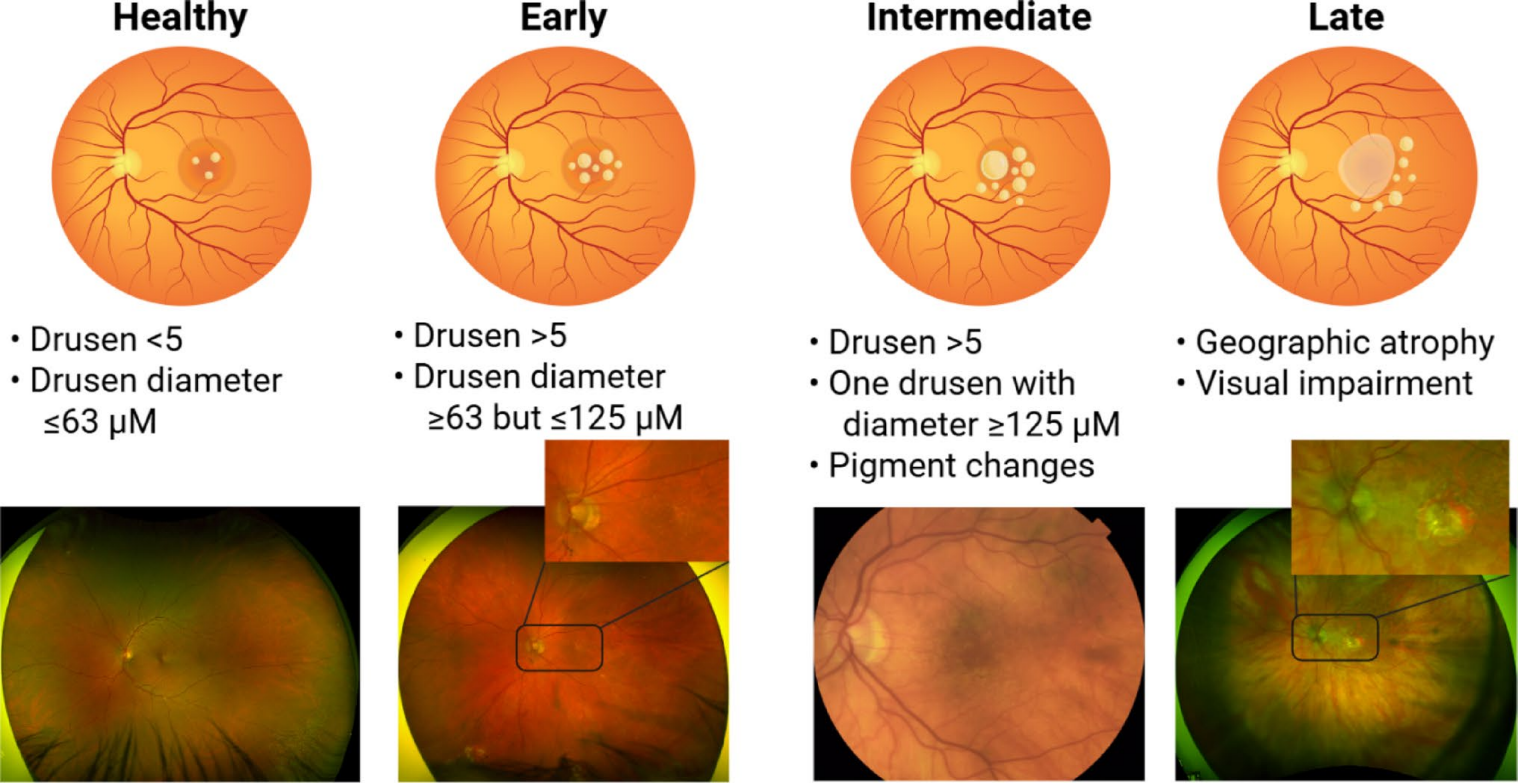
Stages of dry age-related macular degeneration. Dry AMD is categorized into four categories: Healthy, Early, Intermediate, and Late (also known as geographic atrophy). Healthy retinas have few to no drusen, all below 63 µm. Early DAMD has several drusen, of an intermediate size. Intermediate DAMD is characterized by several drusen, with at least one being of a size over 125 µm. Late DAMD is when geographic atrophy and visual impairment begins, which only worsens with time

DAMD is named after the absence of neovascularization and plasma leakage, which are defining characteristics of wet AMD. DAMD is a progressive disease typically tracked through the appearance and number of retinal drusen, yellowish lipid and protein deposits that form beneath the Retinal Pigment Epithelium (RPE) [14–16]. Drusen are hypothesized to be metabolic byproducts and cellular waste, and are associated with the early stages of AMD [17, 18]. DAMD is divided into four clinical stages: Healthy, Early, Intermediate, and Late [19, 20] (Fig. 1). A healthy individual may still have drusen; however, these drusen are small in number, typically fewer than five total, and ≤ 63 μm in diameter. Early DAMD is diagnosed by the presence of a significant amount of drusen between 63 and 125 μm in diameter. Intermediate DAMD is characterized by the presence of at least one drusen above 125 μm in diameter and the start of pigment abnormalities in the retina. Alterations in pigment levels indicate a changing retinal landscape; hyperpigmentation reflects the accumulation of drusen, while hypopigmentation reflects retinal damage and geographic atrophy. In the intermediate stage, visual abnormalities may begin to appear, with some patients remaining asymptomatic while others show signs of blurry and distorted vision [21, 22]. Late DAMD is characterized by geographic atrophy of the fovea without neovascular abnormalities and typically involves corresponding visual impairment [23].

## Cellular characterization of dry age-related macular degeneration

**Fig. 2:**
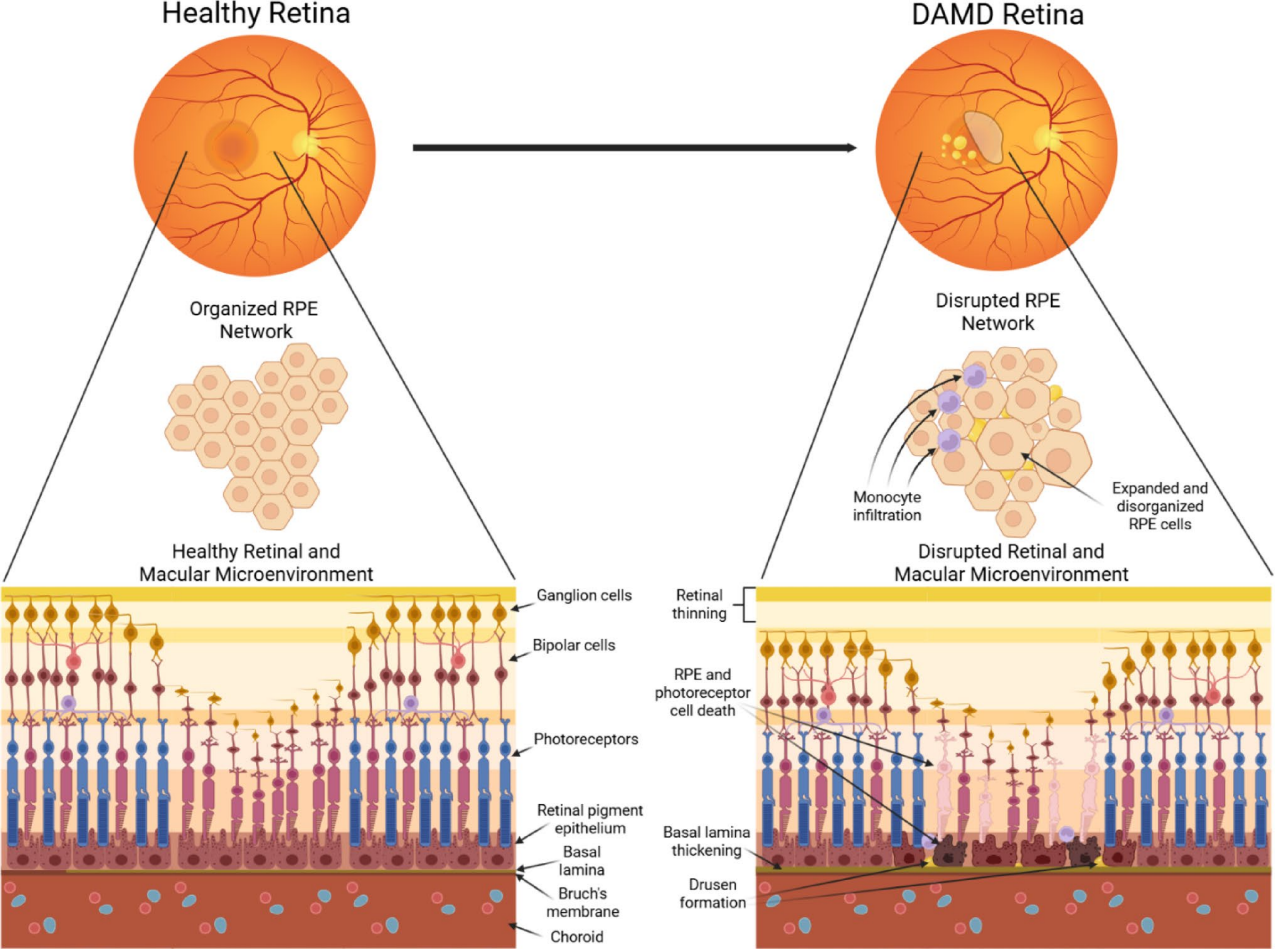
Cellular pathology alterations in dry age-related macular degeneration. A healthy retina contains an organized RPE network with a healthy retinal environment. As disease pathology begins, the RPE network becomes disordered as cells die, drusen disrupt junctions, and monocytes infiltrate the retina. The healthy retinal environment begins to thin with age and the basal lamina thickens, preventing nutrient and waste exchange, resulting in RPE and photoreceptor damage and cell death.

On a cellular level, a major factor in the progression of DAMD is RPE dysfunction. The RPE is a critical player in retinal metabolism and photoreceptor support, serving as the connection between the retina and the choroid for the transfer of metabolites and waste across the blood-retina barrier. As the RPE is a non-dividing cell type, the loss of RPE is permanent, inevitably leading to photoreceptor cell death and visual abnormalities, including central vision loss [24–27]. Young, healthy RPE are packed into neat hexagons. In the early stages of DAMD, the basal lamina of the RPE begins to thicken, generating basal lamina deposits (BLamD) and disrupting RPE interactions with the choroid [28]. The appearance of drusen, typically following the formation of BLamD, further disrupts RPE organization, placing both mechanical and physiological stress on the surrounding RPE cells, which results in cell death [29]. The hexagonal pattern of RPE cells is disrupted as RPE cells die, and the survivors, lacking proliferative ability, must expand to fill the area [30]. Critical levels of cell death lead to geographic atrophy in the macula, with large swaths of the macula lacking any retinal cells, resulting in central vision loss [31] (Fig. 2).

On a structural level, the RPE extends microvilli to support and stabilize the specialized structures of photoreceptors, acting as their support system [32, 33]. As these structures are so closely entwined, dysfunction in the RPE is associated with damage to the photoreceptors [34]. Maeda et al. found that direct damage to the RPE through ornithine treatment caused apoptosis in nearby photoreceptors [35]. Ex vivo retinal TUNEL staining for apoptotic cells showed elevated levels of TUNEL-positive RPE and photoreceptors near areas of atrophy, suggesting these cells’ fates are intertwined [36]. Photoreceptor damage, as measured by Borrelli et al. using ellipsoid zone reflectivity, was increased in patients with intermediate AMD [37].

Interestingly, there is evidence that photoreceptor dysfunction and apoptosis precede RPE dysfunction [33]. Curcio et al. found a 30% decrease in rod abundance as aging occurs, prior to the onset of any disease pathology [38, 39]. Additionally, photoreceptor segment thinning, a process involving a shrinking of the photoreceptor retinal layer, occurs as early as age forty and progresses linearly with age [40]. This process is hypothesized to be due to early dysregulation of cholesterol homeostasis and hypoxic conditions in the retina, which may contribute to the formation of early drusen. This suggests that the molecular pathology of DAMD may begin earlier than previously assumed.

Immune dysregulation also contributes to DAMD pathology. Monocytes play a central role in the pathogenesis of DAMD, particularly in the formation of drusen and disease progression. In atrophic AMD, CCR2⁺ monocytes infiltrate the subretinal space and are associated with photoreceptor degeneration in murine models [41]. In late DAMD, functional alterations in monocytes are influenced by the P2X7 receptor, which is enriched on monocytes and facilitates phagocytosis through its scavenger receptor activity [42]. Reduced membrane fluidity, a downstream effect of P2X7 signaling, is observed across leukocyte subsets, including monocytes, in late AMD. Impaired cholesterol efflux in monocytes further exacerbates disease pathology, promoting the accumulation of cholesterol-rich drusen beneath the RPE in mouse models [43]. These drusen deposits not only characterize AMD but also contribute to its progression, positioning monocyte-driven lipid dysregulation as a potential therapeutic target.

As DAMD is a burgeoning vision crisis for the elderly, there has been considerable research into the pathology and progression of the disease to find biological targets and develop treatments to prevent RPE cell death and ameliorate vision loss. Studies in humans suggest that four major factors contribute to AMD susceptibility: age, family history of AMD, elevated BMI, and cigarette smoking [44–47]. A pillar of AMD research stems from the National Eye Institute’s Age-Related Eye Disease Studies (AREDS and AREDS 2), which began in 1992 and 2006, respectively. These studies followed patients with and without AMD for five years, observing disease progression and response to dietary supplementation [48, 49]. Supplementation with an antioxidant cocktail, including vitamins C and E, beta-carotene, and zinc, delayed the progression of late-stage DAMD and inhibited the progression of DAMD into wet AMD [50]. Unfortunately, there was no improvement among those with early DAMD, suggesting that antioxidant supplementation may not be able to slow or prevent the progression of AMD.

Since AMD is heritable, genetic studies have focused on identifying possible mutations, which may provide insight into the physiology of AMD. In a landmark study, Haines et al. found that the Y402H mutation in Complement Factor H (CFH) significantly increases the risk of AMD development [51]. The Complement System is an innate immunological pathway that initiates a signal cascade upon C1 binding to a pathogen. This cascade stimulates inflammation, recruits phagocytes to pathogens, and facilitates bacterial removal by both optimizing bacteria for phagocytosis and disrupting bacterial membranes [52, 53]. CFH surrounds drusen in people with DAMD. The Y402H mutation may prevent the binding of CFH to its regulator molecule, heparan sulfate; thus, it has been suggested that the Y402H mutation enhances DAMD susceptibility by preventing the regulation of retinal inflammation [16]. Although CFH appears to be a viable target for therapeutics, fourteen CFH inhibitors have been tested, with only three reaching phase III clinical trials. Of those, one failed to provide efficacious treatment, and two increase the risk of retinal neovascularization [54]. Further genome-wide association studies have found potential targets for AMD therapy, including other complement factors, the major histocompatibility complex, and Apolipoprotein E (APOE); however, none have provided a breakthrough in DAMD treatment [55, 56]. Several new avenues for study, including mitochondrial metabolism, lipid regulation and homeostasis, and ferroptosis, are emerging as promising research targets for identifying new therapies.

## Metabolism & mitochondria in dry age-related macular degeneration

Metabolism in the retina is a careful balancing act of nutrient and waste exchange between the choroid, RPE, and photoreceptors. Due to their proximity to the choroid, the RPE facilitates metabolite uptake from the choroidal blood vessels and then shuttles essential material, including metabolites, to the photoreceptors. Photoreceptors heavily utilize glycolysis for the rapid energy turnover necessary for vision. Thus, glucose from the choroid is taken up by the RPE and then released into the retinal space for uptake by the photoreceptors [57]. Photoreceptors then release the lactate produced from glycolysis back to the RPE for conversion to pyruvate and the progression of the TCA cycle. RPE cells rely on mitochondria for energy production, performing oxidative phosphorylation and reductive carboxylation, a unique form of mitochondrial energy production that produces TCA cycle intermediates during hypoxic conditions [58–60]. Increased lactate levels decrease glycolytic activity within the RPE, suggesting a feedback loop between active photoreceptors and the RPE to encourage the shuttling of glucose [61].

Studies investigating the alterations of metabolic pathways in DAMD yield varying results; however, some findings are robust across multiple investigations. A review by Hou et al. consistently identifies altered metabolites in DAMD patients, including phenylalanine, citrate, carnitine, and maltose, which suggests that dysregulation of lipid and carbohydrate metabolism is a key factor in DAMD progression [62]. Studies also strongly suggest that an overactive mTOR pathway drives DAMD pathology, with dysregulation in the AMPK and SIRT1/PGC-1α pathways also contributing to metabolic alterations [63–65]. Interestingly, a recent metabolomics study attempted to classify DAMD progression through metabolic endotypes, taking into account retinal function and structure [66]. Fatty acid metabolites, including acylcarnitines and sphingolipids, were associated with later stages of the disease, further supporting the importance of investigating the crosstalk between metabolism and lipid dysregulation in DAMD [67, 68].

Mitochondria play a central role in cellular metabolism, especially in the RPE. As mentioned above, healthy RPE relies upon mitochondrial oxidative phosphorylation to conserve and shuttle glucose to photoreceptors. Mitochondrial activity is crucial for RPE health, as mice deficient in key mitochondrial proteins exhibit enhanced photoreceptor degeneration [69–71]. As aging occurs, mitochondrial activity naturally decreases; however, mitochondria from individuals with DAMD demonstrate decreased function compared to age-matched controls [72, 73]. Mitochondrial function is significantly decreased in RPE cells isolated from the retinas of patients with AMD compared to those of controls [74, 75]. This is corroborated by electron microscopy (EM) studies, which show a decrease in mitochondrial mass, cristae structure, and electron density in the mitochondrial matrix in eyes with AMD compared to controls [76, 77]. Despite a decrease in mitochondrial mass, reactive oxygen species (ROS) formation was increased in cultured RPE cells from patients with AMD [74]. Several studies also indirectly support this finding by demonstrating that antioxidant supplementation decreases RPE cell death and slows the progression of DAMD in both in vivo and in vitro settings [78–80].

Mitochondria are not stationary organelles but rather dynamic and mobile cellular components. Mitochondrial localization is highly dependent on cellular polarization and morphology; hepatocytes, chromaffin cells, and T cells demonstrate altered mitochondrial localization that depends on polarization, secretory activity, and activity, respectively [81–84]. The RPE is a polarized cell type, with a basolateral domain binding Bruch’s membrane and an apical domain characterized by cilia and direct contact with the photoreceptors [85]. Mitochondria are traditionally thought to accumulate at the basolateral aspect of the RPE, where bioenergetic processes are required; however, modern studies show that this may be due to fixation-based artifacts that alter mitochondrial localization [86–89]. Recent studies demonstrate the presence of apical mitochondria, which may contribute to maintaining the RPE-photoreceptor connection and cooperation [86, 90, 91]. Interestingly, mitochondrial location may contribute to the polarized nature of RPE cells. Hazim et al. found that mitochondrial bioenergetic activity drove RPE differentiation and health in Adult retinal pigment epithelial cell line-19 (ARPE-19) and primary human RPE cells [92].

Mitochondrial DNA (mtDNA) may also play an integral role in the pathology of DAMD. As mitochondria are theorized to have originated from endosymbiotic purple non-sulfur bacteria, their DNA is reminiscent of bacterial DNA. mtDNA is a circular genome of ~ 16,500 bp, encoding 13 polypeptides, ribosomal and tRNA machinery, and several mitochondrial-derived peptides [93–95]. mtDNA lacks the robust repair mechanisms that nuclear DNA (nDNA) possesses, making it more susceptible to mutation and degradation due to natural aging and exogenous stressors [96, 97]. mtDNA mutations, lesions, and fragmentation are well-documented in the retinas of DAMD sufferers, suggesting a connection between mitochondrial dysfunction and DAMD progression [27, 98, 99]. Interestingly, patients with the nDNA CFH allele associated with high DAMD risk demonstrated elevated mtDNA mutation rates, strengthening the hypothesis that CFH dysfunction may contribute to DAMD progression through inflammatory stress and mitochondrial damage [100].

Like nDNA, mtDNA exhibits conserved mutations and single-nucleotide polymorphisms (SNPs), which have been transmitted matrilineally throughout history [101]. These conserved mutations are known as mtDNA Haplogroups (mtDNA-HG) and are associated with susceptibility to certain mitochondrial diseases, including Leber hereditary optic neuropathy, as well as non-metabolic diseases such as Parkinson’s disease [102–107]. There is emerging evidence that mtDNA-HG plays a role in the prevalence of DAMD; individuals in the J haplogroup demonstrate increased susceptibility to DAMD, while those in the H haplogroup appear to be resistant to the disease [108–111]. Despite this, there is disagreement among these studies, with mtDNA-HG disease associations being revised by new studies that utilize more advanced analysis methods and larger sample sizes [112–114]. While it is important to note that concerns exist regarding mtDNA-HG association studies, currently, there is concordance on the association between DAMD susceptibility and resistance, lending credence to the idea that mtDNA influences DAMD progression and pathology.

mtDNA is also responsible for producing mitochondrial-derived peptides (MDPs), a class of pleiotropic polypeptides that may also play a role in DAMD. MDPs range in size but are generally below 50 amino acids in length; this includes the 16-amino-acid mitochondrial open reading frame of the 12 S rRNA type-c (MOTS-c), the 24-amino-acid humanin (HN), and the small-humanin-like peptides 1–6 (SHLP 1–6), which are between 20 and 38 amino acids [115–118]. While their role in DAMD is still unclear, under in vitro settings, MDPs act to protect RPE cells from cell death pathways. HN binds to BAX, preventing mitochondrial localization and the initiation of apoptotic cascades [119, 120]. Of the SHLP family of MDPs, SHLP2 has been investigated for its ability to prevent ROS-induced apoptosis [121]. MOTS-c acts at low concentrations to improve RPE cell viability and mitochondrial biogenesis and function [122].

Mitochondria also play a significant role in lipid metabolism, as lipids are processed through the TCA cycle within the inner mitochondrial membrane. L-carnitine (LC) is a mitochondrial lipid shuttling protein that facilitates the transport of fatty acid chains into the mitochondrial matrix to promote fatty acid β-oxidation; it accomplishes this by converting acyl-coenzyme A esters to acyl-carnitine esters bound to fatty acids, allowing for lipid transport into the mitochondria through Carnitine acyl-carnitine translocase (CAT) [123, 124]. LC also aids in removing intermediate metabolites by binding them and transporting them outside the mitochondria for degradation [125]. LC acts as an anti-inflammatory agent and has been utilized for exploratory supplementation studies to ameliorate inflammation-driven diseases, including AMD [126–128]. When supplemented with ω-3 Polyunsaturated fatty acids (PUFAs) and Coenzyme Q10, LC is effective in increasing visual acuity and slowing disease progression in three separate studies on AMD, ranging from small studies involving 14 and 106 patients to an extensive population study of 400,000 Hungarian patients [129–131]. Additionally, the carnitine shuttle pathway is elevated in all forms of AMD, being moderately upregulated in DAMD but heavily upregulated in wet AMD, with several carnitine isoforms being upregulated at different stages of AMD [132, 133]. This provides an interesting link between mitochondrial dysfunction, lipids, and DAMD pathology.

## Lipid and dry age-related macular degeneration

Lipids play a crucial role in the development of DAMD, although their role in this process will benefit from further study. Genome-wide association studies conducted during the AREDS studies found that lipid pathways, including digestion, mobilization, and transport, are dysregulated in patients with AMD [56]. Notably, the cholesterol pathway, including the APOE, ABCA1, LIPC, and CETP genes, is associated with DAMD risk [134, 135]. The cholesterol pathway is significant as elevated cholesterol levels are indicative of AMD pathology in several animal and in vitro models [136–138]. Indeed, the AREDS study found that many lipid classes are associated with an increased risk of early and late DAMD; specifically, cholesterol, saturated fatty acids, monounsaturated fatty acids, α-Linolenic Acid (ALA), and oleic acid are substantially associated with AMD risk in all forms [139]. Additionally, methylation of the *Elovl*2 gene, which encodes a protein that converts 22-carbon to 24-carbon fatty acids, is elevated with aging; its dysregulation results in an AMD-like phenotype in RPE cells [140–142]. Interestingly, mitochondrial rescue ameliorates this phenotype, lending credence to the idea that there is an intersection between mitochondrial dysfunction, lipid metabolism, and the progression of AMD [142]. The mTOR pathway, closely linked to mitochondrial function, may drive increased fatty acid content, with the IL-17 pathway linking lipid metabolism and the accumulation of lipid droplets [63, 143].

PUFAs are particularly interesting when studying AMD pathology, including ω-3 and ω-6 PUFAs [144–148]. Long-chain PUFAs (LCPUFAs) are essential for maintaining membrane fluidity and are found abundantly in photoreceptors, where they are suggested to interact with rhodopsin to facilitate vision [144, 148, 149]. Research into ω-PUFAs has found that ω-3 PUFAs are generally anti-angiogenic and anti-inflammatory, with a high intake of dietary ω-3 PUFAs associated with a decreased prevalence and progression of AMD [145, 147, 148, 150]. In contrast, ω-6 PUFAs are associated with pro-angiogenic and pro-inflammatory phenotypes, with elevated ω-6 LCPUFA levels found in human eyes diagnosed with AMD compared to controls [145, 146]. ω-3 and ω-6 PUFAs cannot be sufficiently synthesized within the human body. Therefore, dietary supplementation is required to maintain adequate biological levels [151]. As such, supplementation studies utilizing ω-3 PUFAs have been implemented, although no changes in visual acuity or disease progression were seen in these studies [147].

Not all lipids provide protective effects from AMD [67, 68]. As mentioned above, high ALA intake is positively associated with diagnosis and poor prognosis in all forms of AMD [139]. ALA is an essential 18-carbon ω-3 PUFA with three conjugated double bonds commonly ingested through nuts, fruits, and oils [152, 153]. Interestingly, although ALA consumption is positively associated with AMD diagnosis and progression, ALA is protective in several other diseases, including cardiovascular disease, hypertension, and breast cancer due to its anti-inflammatory properties [154–157]. It is surprising, then, that it negatively impacts AMD and may have a significant inflammatory component in its progression [158–161]. Recent studies demonstrate that ALA and other linolenic acids can induce ferroptosis in various cell types, uncovering a previously underexplored mechanism by which these fatty acids may contribute to AMD progression [162–165].

Lipid peroxidation is another pathway of study within DAMD pathology. Peroxidation occurs when lipids containing double bonds are attacked by ROS, which can result in conformational changes and the addition of oxygen [166, 167]. PUFAs, specifically, are common targets of ROS for lipid peroxidation, as the abundance of double bonds is an easy target for electrophilic ROS [168]. Multiple studies find that plasma lipid peroxidation levels are elevated in patients with DAMD, with the levels of peroxidation correlating positively with disease severity [169–171]. A 2012 study demonstrated that CFH Y402H, mentioned above as conferring an increased risk of AMD development, binds peroxidized phospholipids and initiates complement activity, suggesting a connection between the long-studied complement system and lipid peroxidation [172, 173].

Patients with DAMD also exhibit elevated plasma levels of 2-(ω-carboxyethyl)pyrrole (CEP), a protein adduct formed from the free-radical oxidation of docosahexaenoate (DHA)-containing lipids [174]. This is consistent with elevated CEP levels in the retina and drusen of patients with DAMD; as photoreceptors contain abundant DHA levels, this suggests that the elevated retinal accumulation of lipid peroxidation is shuttled from the RPE into the blood or, failing that, stored within extracellular drusen for later processing [175–177]. Interestingly, cones contain lower concentrations of DHA than rods, though cones are typically the most affected by RPE pathology; this implies that rod lipid dysfunction may drive AMD pathology, while cones bear the brunt of the consequences [178].

In animal models of DAMD, there is significant evidence that aberrant lipid peroxidation contributes to DAMD pathology. ARPE-19 cells supplemented with DHA and lipofuscin and exposed to blue light irradiation lead to decreased glutathione levels, mitochondrial membrane potential, and cell viability [179]. Ex vivo mouse retinas exposed to 4-hydroxynonenal (4-HNE) induce RPE cell death through increased lipid peroxidation [180]. A mouse model that utilized immunization with CEP to drive a DAMD pathology developed drusen and RPE lesions mimicking those seen in humans with DAMD, a phenotype not naturally observed in mice [181]. These CEP-immunized mice developed greater retinal macrophage involvement than controls; these macrophages were also polarized to the proinflammatory M1 phenotype [182]. Taken together, this demonstrates that reactions to peroxidized lipids may initiate a cascade that leads to DAMD pathology. Additionally, a knockout of glutathione peroxidase 4 (GPx4), an antioxidant enzyme that reduces oxidized lipids, in adult mouse retinas results in RPE cell death, photoreceptor thinning, and a loss of mitochondrial integrity; an elevated level of lipid byproducts, including CEP and 4-HNE, accompanied this [183, 184]. Interestingly, GPx4 is a key enzyme in ferroptosis, a cell death mechanism that depends on iron accumulation and lipid peroxidation [185, 186].

## Ferroptosis and dry age-related macular degeneration

Ferroptosis, a biological mechanism defined by Dixon et al. in 2013, has since been investigated for its roles in cancer regulation, neurodegeneration, and ischemia-reperfusion injuries [185, 187]. In a broad sense, ferroptosis occurs through the buildup of ROS, which may stem from the iron-dependent Fenton reaction, mitochondrial dysfunction, or other stressors. As ROS production increases, antioxidant defense begins to fail, leading to the accumulation of lipid peroxides [188, 189]. Lipid peroxidation causes destabilization of cellular and organellar membranes, resulting in cell death.

As denoted by the name, ferroptosis is an iron-dependent mechanism of cell death. There is significant evidence that elevated iron levels, in circulation and within the retina, contribute towards the development and progression of DAMD pathology [190]. As aging occurs, iron levels in the retina tend to increase; however, people with DAMD exhibit higher levels of serum iron, retinal iron, and ferritin, an iron-sequestering protein, compared to healthy controls, implying disrupted iron homeostasis [191–193]. This is mirrored in aged mice, which expressed significantly increased iron levels and ferritin mRNA expression [194]. Transferrin, an iron-transport protein, also exhibited elevated mRNA and protein expression in retinas from individuals with DAMD, thereby increasing the evidence that iron dysregulation contributes to DAMD pathology [195]. Interestingly, a recent study by Qu et al., analyzing serum iron status biomarkers in nearly 24,000 European individuals, found that DAMD pathology was positively associated with transferrin levels and negatively associated with ferritin levels [196]. However, the ferritin association did not fully meet statistical significance (*p* = 0.074). Taken together, the association between retinal iron dysregulation and DAMD has bolstered interest in determining the role of ferroptosis in DAMD.

Multiple studies implicate ferroptosis and oxidative pathology as key drivers of AMD pathology, using both in vitro and in vivo studies [197–203]. Several key proteins, including low-density lipoprotein receptor, pigment epithelium-derived factor, and heme oxygenase-1 (HO-1), are implicated as drivers of ferroptosis in the retina [197, 198, 201]. HO-1 expression is increased by a buildup of all-trans-retinal (ATR), the product of 11-cis-retinal becoming photoisomerized in the photoreceptor [204, 205]. The accumulation of ATR appears to drive ferroptosis through HO-1, as ATR-induced pathology is ameliorated through both inhibition of ferroptosis, through ferrostatin-1, and HO-1 inhibition, through zinc protoporphyrin IX and HO-1 knockdown [204, 206]. Additionally, a blue light-induced model of murine AMD exhibits elevated iron levels, HO-1 expression, and RPE cell death, accompanied by reduced GSH and GPx4 levels, indicating a ferroptotic phenotype [199, 200]. These results were confirmed in an ARPE-19 immortalized RPE cell line [199]. A mouse model of a high-fat Western diet developed spontaneous RPE dysfunction, characterized by elevated retinal iron levels, HO-1 expression, and lipid peroxidation [207]. Interestingly, this work demonstrated that IL-1β, a pro-inflammatory cytokine, was required for this process [207]. As monocytes are a primary source of IL-1β production, this further supports the hypothesis that aberrant monocyte lipid peroxidation may contribute to DAMD pathology through a ferroptotic process [208].

Lipocalin-2 (LCN2) is a circulatory adipokine with antibacterial and anti-inflammatory activity [209]. It accomplishes this by binding bacterial siderophores to prevent bacterial iron accumulation and by limiting autophagic responses to stress [210–213]. Gupta et al. identify LCN2 as a critical regulator of RPE degeneration in DAMD [214, 215]. Using a mouse model, the study demonstrates that increased LCN2 expression in RPE cells impairs autophagy while simultaneously activating inflammasome signaling and ferroptosis. This coordinated disruption leads to chronic inflammation and lipid peroxidation, ultimately resulting in the progressive death of RPE cells. These findings position LCN2 as a mechanistic link between lipid peroxidation, defective autophagy, and ferroptotic stress in DAMD, suggesting that targeting LCN2 or its downstream pathways may offer therapeutic benefits.

Finally, analyzing gene expression data from nearly 100 human eye tissue samples revealed ten differentially expressed ferroptosis genes in eyes with AMD, including VEGFA, HAMP, SLC2A1, and FADS2 [216]. SLC2A1 and FADS2 expression elevation was further confirmed in a sodium iodate-induced AMD mouse model [216]. Altogether, there is significant evidence that ferroptosis plays a role in DAMD pathology, though little has been done to examine the role of mitochondria in this process.

## Discussion

While ferroptosis, lipid dysregulation, and peroxidation are intrinsically linked, the role of mitochondria in the progression of ferroptosis remains a contentious issue. Mitochondrial morphology is significantly altered during ferroptosis, characterized by a decrease in mitochondrial size and an increase in mitochondrial density [185, 217, 218]. Additionally, the mitochondrial protein Lon Peptidase 1 (LONP1) is implicated in susceptibility to ferroptosis [186, 219]. Despite this, studies also demonstrate a mitochondria-independent mechanism for ferroptosis. Early work on ferroptosis revealed that mitochondrially depleted ρ0 cells remained susceptible to erastin-induced ferroptosis; however, subsequent studies found that mitochondrial depletion through Parkin overexpression protected cells from a similar erastin-induced ferroptosis [185, 220]. More recent work by Oh et al. demonstrated that SK-Hep1 ρ0 cells were more resistant to ferroptosis than their parental SK-Hep1 ρ + cells, suggesting that mitochondria play a crucial role in ferroptosis [221]. Therefore, it remains of interest to elucidate the role of ferroptosis and mitochondria in AMD, as these factors may work independently or in conjunction to influence disease progression. To this end, we will review the three regulatory mechanisms for ferroptosis, all of which are linked to mitochondrial function, the GSH/GPx4 pathway, iron metabolism, and lipid metabolism and peroxidation [222].

As mentioned above, GPx4 is an antioxidant enzyme that ameliorates lipid peroxidation. This process involves converting lipid peroxides into their corresponding alcohols, which requires glutathione (GSH) to be oxidized into glutathione disulfide (GSSG) [223, 224]. GSH is a required substrate for this reaction and is heavily utilized in the cell for its radical scavenging properties [225]. GSH synthesis requires cysteine, which is taken up by cells through the system Xc- amino acid antiporter [222]. As cells lack redundancies in these pathways, the inhibition of GPx4, system Xc-, and glutathione synthetase, as well as cysteine depletion, all induce ferroptosis through the buildup of lipid peroxides [222, 226]. As the primary site of cellular ROS production, GSH is crucial for mitochondrial function, with several studies characterizing mitochondrial dysfunction following GSH depletion [227, 228]. Additionally, cysteine is essential for iron-sulfur (Fe-S) complexes, which are utilized in the electron transport chain (ETC); this will be discussed later in the context of iron metabolism in the mitochondria [229].

Iron is a required element for many cellular processes, including energy metabolism; therefore, iron metabolism is typically tightly controlled by the cell [230]. Iron is generally bound to the transferrin receptor protein, which regulates iron uptake in a pH-dependent manner, then endocytosed into the cell, though other metal transporters also play a role in cellular uptake [231]. Once inside the cell, iron is released into the labile iron pool, a source of free iron for use by multiple cellular processes [232]. Free iron in its Fe2 + form is capable of undergoing the Fenton reaction, converting peroxides into hydroxyl radicals, which can go on to oxidize lipids [233]. This reaction produces considerable ROS, quickly overwhelming cellular defenses, including GPx4, resulting in ferroptosis. Iron level is a key player in ferroptosis, with excessive iron contributing to increased susceptibility to ferroptosis [185, 234]. Therefore, the labile iron pool is generally quickly trafficked to its destinations, sequestered into ferritin, a heteropolymer capable of self-assembling into an iron-containing shell, or exported from the cell [231, 235].

One of the main destinations for intracellular iron is the mitochondria, where it is utilized for heme and Fe-S complex synthesis [229, 236]. Mitochondria make extensive use of heme- and Fe-S complex-containing enzymes following biosynthesis. The ETC, in particular, involves heme and Fe-S complexes in Complexes I–IV, with ATP synthase being the only enzyme that does not have an iron cofactor [237, 238]. Heme is a tetrapyrrole structure, meaning a quartet of pyrrole rings linked cyclically, with a centralized iron atom, and acts as a prosthetic group for numerous enzymes [239–241]. Heme synthesis is initiated in the mitochondrial matrix with succinyl-CoA and glycine converted into 5-aminolevulinic acid by 5-aminolevulinate synthase [242]. 5-aminolevulinic acid is transported out of the mitochondria for several further steps before returning for final biosynthesis, which involves the insertion of iron by ferrochelatase [242]. Fe-S complexes are a common form of protein cofactor that exists in numerous structural isoforms, including 2Fe-2 S rhomboids, 4Fe-4 S cubes, and 3Fe-4 S semi-cubic forms [238]. Interestingly, these Fe-S complexes are typically anchored to cysteine amino acids, where a disulfide bond firmly anchors the cofactor to the protein [243, 244]. Thus, a depletion of cysteine through GSH synthesis could elevate free iron levels by indirectly preventing iron sequestering into Fe-S complexes.

As phospholipids and their peroxidation are a key feature of ferroptosis, their metabolism may regulate ferroptotic susceptibility and progression. Phosphatidylcholine (PC) and phosphatidylethanolamines (PE) containing arachidonic acid (AA) are PUFAs preferentially targeted for oxidation during ferroptosis [216, 245, 246]. AA is a common modification to PC and PE species through the activity of acyl-CoA synthetase long-chain family member 4 (ACSL4), which binds AA to coenzyme A, and lysophosphatidylcholine acyltransferase 3 (LPCAT3), which preferentially binds AA-CoA to PC and PE species [246–248]. Interestingly, Doll et al. found that ACSL4 regulated ferroptosis sensitivity through a CRISPR-based genetic screen, while Dixon et al. identified both ACSL4 and LPCAT3 as important proteins in ferroptosis through an insertional mutagenesis study [217, 249]. Combined, these studies suggest that AA is a critical PUFA for ferroptosis progression. AA, while typically ingested through meat and animal products, can also be synthesized through linoleic acid (LA) [250, 251]. Of interest for DAMD is that the AREDS studies found that elevated LA levels were significantly positively correlated with late AMD and geographic atrophy [139]. The data surrounding AA levels is more mixed, with AREDS finding a weak correlation between AA levels and AMD progression, AREDS 2 finding a minimal to negative correlation, and another study finding that people with AMD had higher levels of plasma AA [139, 252].

PC and PE are the most abundant phospholipids in the mitochondrial membrane, thereby increasing the organelle’s susceptibility to lipid peroxidation [253, 254]. Interestingly, the ratio of PC to PE may influence mitochondrial efficiency [255]. PE *N*-methyltransferase (PEMT) is responsible for PC synthesis from PE, and modulation of this protein can alter lipid concentrations in organelle membranes [256]. PEMT^−/−^ mice showed a ~ 220% decrease in PC levels in mitochondrial membranes and a corresponding increase in PE. PEMT^−/−^ mice showed greater hepatic mitochondrial respiration and ATP production [255]. In contrast, PE-depleted mitochondria demonstrate decreased respiratory capacity and ATP production [257]. Alterations in PC concentrations in the mitochondrial membrane may also disrupt mitochondrial importer proteins TIM22 and TIM23, thereby aggravating pathological conditions [258].

## Conclusion

While the exact role of mitochondria and their critical role in ferroptosis is still under debate, there is clear evidence that ferroptosis, mitochondria, and lipids are intrinsically linked. These factors are also closely related to DAMD development and pathology, as described above. Given these potential links, it may not be surprising that therapies targeting only mitochondrial rescue are currently ineffectual in halting DAMD progression. Elamipretide is a small peptide molecule that acts to stabilize mitochondrial inner membrane architecture through interactions with cardiolipin [259, 260]. Two clinical trials, ReClaim I and II, demonstrated mild efficacy of elamipretide in attenuating intermediate DAMD progression, as evidenced by gains in best-corrected visual acuity (BCVA); however, the rate of adverse events was particularly high in both trials [261, 262]. Risuteganib is an oligopeptide that inhibits integrin receptors to decrease inflammation and mitochondrial dysfunction [263]. Risuteganib showed consistent positive results in Phase I and II trials, with BCVA levels increasing by five to ten letters [264–267]. Despite these gains, no drug on the market can entirely prevent or halt the progression of DAMD, perhaps because they are targeting only one component of a cooperative system. Mitochondrial rescue can only slow ferroptosis if it does not target the underlying mechanisms by which ferroptosis progresses.

**Fig. 3:**
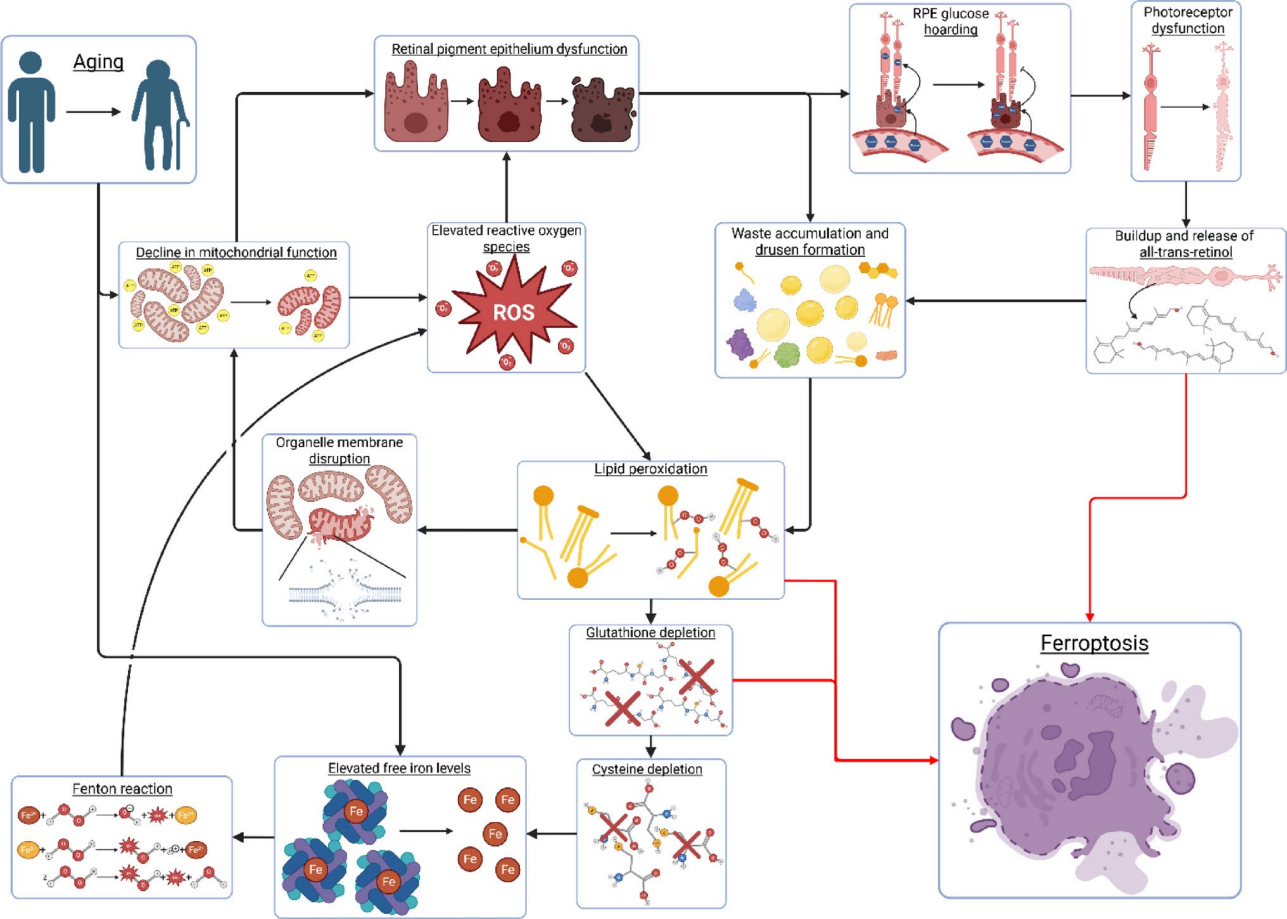
A proposed mechanism for natural aging processes setting off a vicious cycle leading towards ferroptosis. Aging results in a natural decline in mitochondrial function. In a retinal context, this results in RPE cells declining in function and producing ROS. This, coupled with photoreceptor dysfunction, results in an accumulation of waste and drusen formation. As waste and ROS accumulates, lipids begin to become modified with peroxides. GPx4 utilizes glutathione to prevent lipid peroxidation, however this depletes cysteine levels and begins to release free iron as iron sulfur complexes require cysteine for synthesis. As free iron levels increase, the Fenton reaction initiates even more ROS production, worsening lipid peroxidation. Lipid peroxidation can outpace GPx4 prevention, leading to mitochondrial membrane disruption and worsened mitochondrial function, continuing the cycle. Throughout this cycle, lipid peroxidation, the release of all-trans-retinol from photoreceptors, and glutathione depletion all contribute to the ferroptosis process

This leads to the hypothesis that the intersection of these cellular processes, coupled with inflammatory processes such as those associated with the complement system, may drive pathology in DAMD (Fig. 3). Mechanistically, initial pathology may begin with the natural aging process. As aging progresses, a natural decline in mitochondrial function occurs, accompanied by an increase in ROS production [268]. RPE cells begin to favor glycolytic metabolic function and shuttle less glucose to the photoreceptors. A decrease in photoreceptor energy availability initiates thinning of the photoreceptor layer and a buildup of byproducts. This includes ATR, which may induce HO-1 expression and begin early ferroptotic processes. The RPE, already deficient in energy, fails to properly digest waste byproducts, both internal and from the photoreceptors, resulting in sequestration on Brusch’s membrane and drusen formation. As waste products and ROS accumulate, PUFAs are susceptible to peroxidation, destabilizing organellar and cellular membranes. Individuals with high fatty acid intake, including LA, ALA, and AA, may be more vulnerable to pathology, with ALA inducing further ferroptotic processes and LA and AA inducing higher levels of modified PC and PE, which are susceptible to peroxidation. GPx4 and GSH are set to work eliminating lipid peroxidation, but they consume cellular cysteine reserves. The depletion of cysteine results in an elevation of free iron, as Fe-S complex synthesis is halted. This free iron, already elevated in older individuals, may then proceed to generate increased ROS through the Fenton reaction, exacerbating the ROS stress load. Indeed, oxidative stress is capable of inducing mitochondrial iron overload and ferroptosis in cardiomyocytes [269]. Lipid peroxidation begins to build in PC and PE populations, especially within the mitochondrial membrane. This further inhibits mitochondrial function, setting the cell further down on this vicious cycle, which ultimately leads to cell death through ferroptosis. The loss of RPE cells is irreversible and triggers inflammatory responses, further exacerbating the system’s stress. Ultimately, as photoreceptors and RPE cells die, clinically relevant pathology becomes obvious. However, the time for intervention may have already passed.

With this proposed mechanism comes the possibility of exploring new hypotheses. To begin, susceptibility to DAMD pathology may be partially driven by mitochondrial lipid composition, as elevated PC and PE levels may facilitate more rapid organelle destabilization. We hypothesize that elevated levels of PC and PE in mitochondria correlate with a higher likelihood of developing DAMD. We also hypothesize that PC and PE levels may decrease following DAMD pathology, as ferroptotic processes may have already pruned the mitochondria and cells most vulnerable. It is essential to determine the lipid composition of retinal mitochondria and to compare the mitochondrial compositions of individuals with healthy eyes and those with DAMD. These insights would facilitate a deeper understanding of risk factors for DAMD.

Second, DAMD pathology may be correlated with a decrease in bioavailable cysteine levels. Several studies have utilized N-acetyl-cysteine to ameliorate ROS-induced damage to primary RPE cells and improve glutathione production; however, none have so far analyzed cysteine levels within the retina of individuals with and without DAMD [270, 271]. We hypothesize that people with DAMD pathology will exhibit decreased retinal cysteine levels compared to healthy controls. Additionally, a decrease in retinal cysteine levels may serve as an early marker for DAMD pathology; however, due to the difficulty in measuring retinal cysteine levels, this finding may not be clinically relevant.

Finally, while elevated iron levels are known to be associated with DAMD pathology, it is not well understood where that iron is sourced. We hypothesize that, during early DAMD pathology, iron levels are elevated through both an influx from the choroid and a release from Fe-S complexes within the retina itself. Therefore, it is prudent to determine the levels of Fe-S containing proteins within the retina with respect to age and disease pathology. This would shed light on how and why iron levels are correlated with DAMD pathology, and perhaps provide insight into new therapeutics to ameliorate high retinal iron levels.

In this review, we present evidence that mitochondrial function, lipids, and ferroptosis are interconnected in DAMD pathology, potentially driving disease progression. We also introduce the hypothesis that natural aging initiates a vicious cycle that exacerbates retinal metabolism, accumulates toxic waste products, and leads to ferroptosis. We proceed to suggest discrete hypotheses to test to elucidate whether our proposed hypothesis holds up to scrutiny by the field. Future testing remains crucial for understanding these cellular processes, testing these hypotheses, and identifying promising targets for DAMD therapies.

## Data Availability

No datasets were generated or analysed during the current study.
